# Supplementary material for: A Global Screen for Assembly State Changes of the Mitotic Proteome by SEC-SWATH-MS
Source: Cell Syst. 2020 Feb 26;10(2):133–155.e6. doi: 10.1016/j.cels.2020.01.001 (PMC7042714; doi:10.1016/j.cels.2020.01.001)

Q7L5Y9 | MAEA_HUMAN | MAEA EMP HLC10 PIG5
Monomer MW [kDa]: 45.287 Monomer expected elution fraction: 47

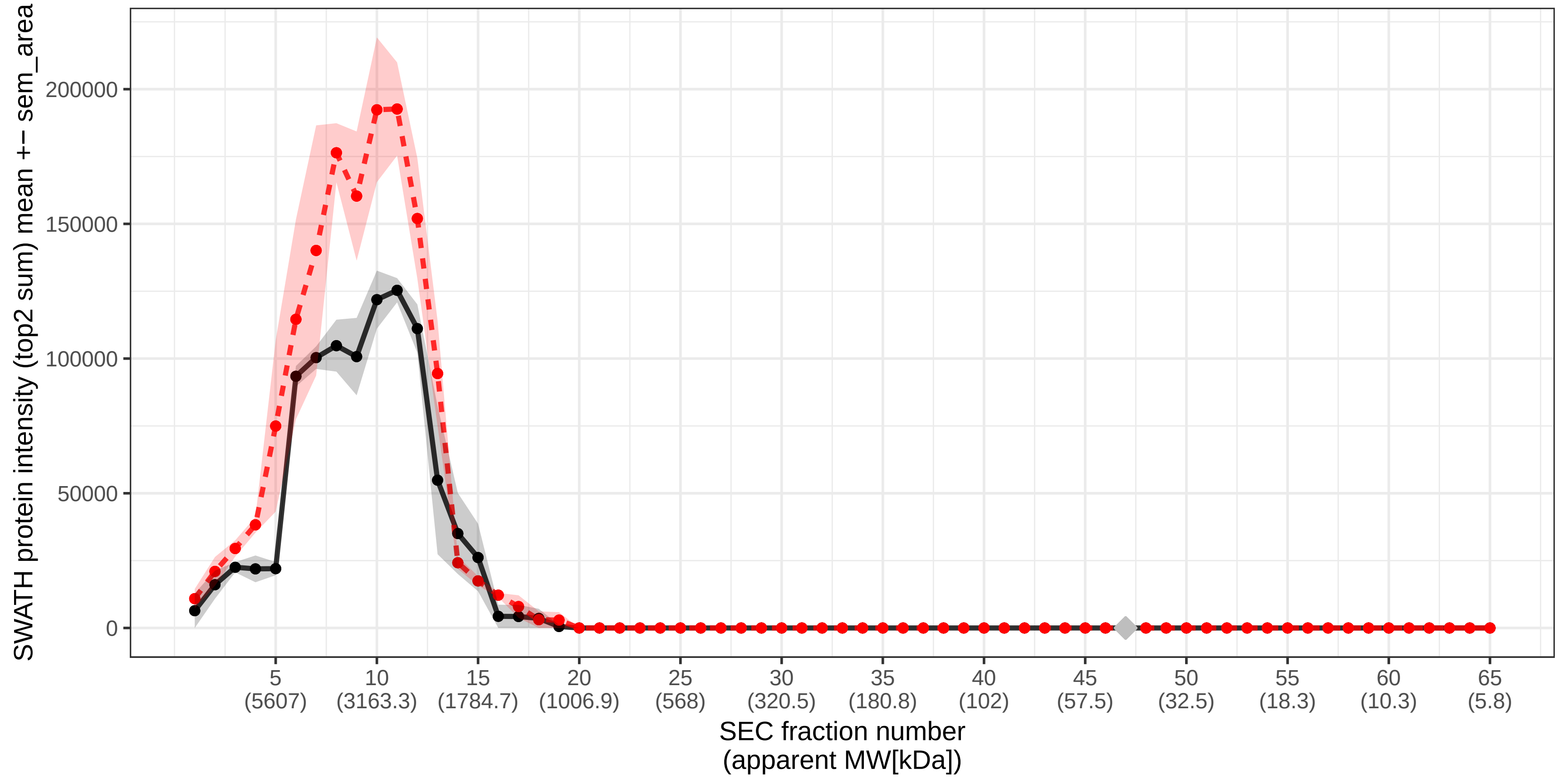

Supplement: Data S1. SEC-SWATH-MS Protein Chromatograms, Related to Figure 1 [file mmc6.zip › SECchrom_Q7L5Y9_MAEA_HUMAN_MAEA_EMP_HLC10_PIG5.pdf]
